# Identification of novel serum autoantibody biomarkers for early esophageal squamous cell carcinoma and high-grade intraepithelial neoplasia detection

**DOI:** 10.3389/fonc.2023.1161489

**Published:** 2023-05-12

**Authors:** Zhibin Chen, Jie Xing, Cuiling Zheng, Qianyu Zhu, Pingping He, Donghu Zhou, Xiaojin Li, Yanmeng Li, Saiping Qi, Qin Ouyang, Bei Zhang, Yibin Xie, Jiansong Ren, Bangwei Cao, Shengtao Zhu, Jian Huang

**Affiliations:** ^1^ Beijing Institute of Clinical Medicine, Beijing Friendship Hospital, Capital Medical University, Beijing, China; ^2^ Beijing Key Laboratory for Precancerous Lesion of Digestive Disease, Department of Gastroenterology, National Clinical Research Center for Digestive Disease, Beijing Digestive Disease Center, Beijing Friendship Hospital, Capital Medical University, Beijing, China; ^3^ Department of Clinical Laboratory, National Cancer Center/National Clinical Research Center for Cancer/Cancer Hospital, Chinese Academy of Medical Sciences and Peking Union Medical College, Beijing, China; ^4^ Department of Pancreatic and Gastric Surgery, National Cancer Center/National Clinical Research Center for Cancer/Cancer Hospital, Chinese Academy of Medical Sciences and Peking Union Medical College, Beijing, China; ^5^ Office of Cancer Screening, National Cancer Center/National Clinical Research Center for Cancer/Cancer Hospital, Chinese Academy of Medical Sciences and Peking Union Medical College, Beijing, China; ^6^ Department of Oncology, Beijing Friendship Hospital, Capital Medical University, Beijing, China; ^7^ National Clinical Research Center for Digestive Disease, Beijing Friendship Hospital, Capital Medical University, Beijing, China

**Keywords:** esophageal squamous cell carcinoma, high-grade intraepithelial neoplasia, serum biomarker, autoantibody, early detection

## Abstract

**Background:**

Early diagnosis of esophageal squamous cell carcinoma (ESCC) is critical for effective treatment and optimal prognosis; however, less study on serum biomarkers for the early ESCC detection has been reported. The aim of this study was to identify and evaluate several serum autoantibody biomarkers in early ESCC.

**Methods:**

We initially screened candidate tumor-associated autoantibodies (TAAbs) associated with ESCC by serological proteome analysis (SERPA) combined with nanoliter-liquid chromatography combined with quadrupole time of flight tandem mass spectrometry (nano-LC-Q-TOF-MS/MS), and the TAAbs were further subjected to analysis by Enzyme-linked immunosorbent assay (ELISA) in a clinical cohort (386 participants, including 161 patients with ESCC, 49 patients with high-grade intraepithelial neoplasia [HGIN] and 176 healthy controls [HC]). Receiver operating characteristic (ROC) curve was plotted to evaluate the diagnostic performance.

**Results:**

The serum levels of CETN2 and POFUT1 autoantibodies which were identified by SERPA were statistically different between ESCC or HGIN patients and HC in ELISA analysis with the area under the curve (AUC) values of 0.709 (95%CI: 0.654-0.764) and 0.741 (95%CI: 0.689-0.793), 0.717 (95%CI: 0.634-0.800) and 0.703 (95%CI: 0.627-0.779) for detection of ESCC and HGIN, respectively. Combining these two markers, the AUCs were 0.781 (95%CI: 0.733-0.829), 0.754 (95%CI: 0.694-0.814) and 0.756 (95%CI: 0.686-0.827) when distinguishing ESCC, early ESCC and HGIN from HC, respectively. Meanwhile, the expression of CETN2 and POFUT1 was found to be correlated with ESCC progression.

**Conclusions:**

Our data suggest that CETN2 and POFUT1 autoantibodies have potential diagnostic value for ESCC and HGIN, which may provide novel insights for early ESCC and precancerous lesions detection.

## 1 Introduction

The esophageal cancers can be fatal, and its subtype, esophageal squamous cell carcinoma (ESCC), accounts for approximately 90% of all esophageal cancers, ranking seventh in cancer incidence and sixth in cancer mortality worldwide (1). The detection rate of early ESCC in China is around 10% because of inadequacies in screening and early diagnostic techniques. Diagnosis is often made at an advanced stage, leading to a 5-year survival rate of <20% (2). ESCC occurrence and development is a multistage process involving precancerous lesions, early and advanced cancer stages (3). Furthermore, identifying early-stage ESCC is key to reducing mortality (4, 5); however, its prevention and treatment remain a global problem.

The monitor of precancerous lesions of ESCC and early ESCC is important for the prevention and prognosis. According to the level of lesion involvement, it was divided into low-grade intraepithelial neoplasia (LGIN, confined to the lower half of the squamous epithelium) and high-grade intraepithelial neoplasia (HGIN, involved more than the lower half of the esophageal squamous epithelium) (6), and early ESCC was considered as tumors limited to the upper two-thirds of the submucosa (7).

The screening methods for early ESCC and precancerous lesions include endoscopic biopsy, noninvasive cytology examination, and imaging. Endoscopy is extremely invasive, requires highly trained medical staff, so that often results in false positives or a high rate of missed detection. Presently, conventional antigenic tumor markers, such as carcinoembryonic antigen (CEA) and carbohydrate antigen (CA) 19-9, show low sensitivity or specificity in varying degrees in the screening and diagnosis of ESCC (8, 9) and the “Guideline for the diagnosis and treatment of esophageal cancer (2022)” (6) clearly stated that clinical ESCC-specific serum tumor markers remained to be established.

Recent studies reported that during tumor development, tumor-associated antigens (TAAs) may emerge during the transition to malignancy. Furthermore, due to changes in the immune status, the antibody immune response against certain autoantigens may produce a large number of autoantibodies (10, 11). These autoantibodies can be easily detected clinically because of their stability and persistence in serum samples (12, 13). Moreover, improvements in antibody detection technology have triggered further interest in the utilization of autoantibodies as diagnostic and prognostic biomarkers of cancer (14). Existing research shows that examining patient serum autoantibodies for screening and early diagnosis has unique advantages and provides strong operability (15, 16).

Here we aimed to screen serum autoantibody biomarkers for the diagnosis of early ESCC based on serological proteome analysis (SERPA) and nanoliter-liquid chromatography combined with quadrupole time of flight tandem mass spectrometry (nano-LC-Q-TOF-MS/MS). The candidate autoantibodies with ESCC-specific immune response were initially screened and then analyzed by enzyme-linked immunosorbent assay (ELISA) for detecting their levels in a clinical cohort (49 patients with HGIN, 82 with early ESCCs, 79 with advanced ESCCs and 176 healthy controls [HC]). Finally, the expression of TAAs in tissues of different ESCC stages was observed by immunohistochemistry to explore its relationship with the production of autoantibodies. Our results suggest that CETN2 (Centrin-2) and POFUT1 (GDP-fucose protein O-fucosyltransferase-1) autoantibodies have potential diagnostic value for early ESCC and precancerous lesions, thereby providing new ideas for detecting early ESCC.

## 2 Materials and methods

### 2.1 Study population and tissues samples

A total of 386 participants comprising 56 patients with ESCC and precancerous lesions and 176 HC collected from Beijing Friendship Hospital, Capital Medical University during 2015–2022, 154 ESCC participants were collected from Cancer Hospital, Chinese Academy of Medical Sciences and Peking Union Medical College from December 2021 to November 2022. ESCC and precancerous tissues comprising 14 patients with intraepithelial neoplasia (IN) and 13 patients with ESCC were obtained from 2008 to 2012 at Beijing Friendship Hospital, Capital Medical University. The demographic parameters of the samples were shown in **Supplementary Table 1**.

All patients had undergone endoscopic biopsy and received a definite pathological diagnosis, HC were eligible blood donors with no evidence of malignancies or immune system disease based on physical examinations. The study protocol was formulated according to the Research Medical Ethics Committee of Beijing Friendship Hospital, Capital Medical University and Cancer Hospital, Chinese Academy of Medical Sciences and Peking Union Medical College. Informed consent was obtained from each patient.

The diagnosis of ESCC was defined according to the Seventh Edition of the American Joint Committee on Cancer (AJCC) Cancer Staging Manual (17). As precancerous lesion, IN was further classified as HGIN and LGIN (18).

All serum samples were stored at −80°C.

### 2.2 SERPA and nano-LC-Q-TOF-MS/MS analysis for screening ESCC-related TAAbs

The cellular proteins were processed as follows: five ESCC cell lines were used to extract total protein i.e., TE-1, TE-8, EC109, KYSE30, and KYSE150. Among them, TE-1, EC109 and KYSE30 were purchased from the Cell Resource Center, Institute of Basic Medicine, Chinese Academy of Medical Sciences (Beijing, China), TE-8 and KYSE150 were provided by the Department of Oncology, Beijing Friendship Hospital, Capital Medical University. All cells were digested and centrifuged at 800 rpm for 5 min, and then five times the volume of lysate buffer was added to it. After three cycles of freezing and thawing in liquid nitrogen, 50 µg/mL RNase and 200 µg/mL DNase were added to the tube and centrifuged at 14000 rpm for 40 min at 4°C following in an ice bath for 30 min. The protein concentration was determined using a 2-D Quant kit (GE, USA), and the extracted protein was purified using a 2-D Clean-up kit (GE). The hydration solution (124 µL) was equilibrated at room temperature (27°C) in advance and 1 µL immobilized pH gradient (IPG) buffer (pH 3–10, GE) was added to the purified protein sample. The mixture was incubated at room temperature for 30 min, shaken once every 10 min, and centrifuged at 14000 rpm for 40 min at 18°C.

SERPA analyses and two-dimensional electrophoresis (2-DE) were performed as previously described (19). Briefly, 100 µg of protein was loaded on a 7 cm, pH 3–10, IPG precast strip (GE) to perform first-dimensional isoelectric focusing (IEF). Next, second-dimensional sodium dodecyl sulfate-polyacrylamide gel electrophoresis (SDS-PAGE) was performed followed by western blot analysis. The protein extracted from SDS-PAGE was transferred to a polyvinyl difluoride (PVDF) membrane (0.45μm pore size, Millipore, USA) at 300 mA for 1 h, and then blocked with prefiltered 5% skim milk (BD, USA) for 3 h at room temperature. Serum samples from 10 patients with early ESCC were randomly selected and mixed as the primary antibody for membrane incubation in cancer group, and serum samples from 10 healthy participants were mixed as the primary antibody for membrane incubation in control group. Separate membranes were used in the cancer group and control group, respectively. The membrane was incubated with serum (dilution: 1:100) overnight at 4°C. After washing six times with 1% Tris-buffered saline with 0.1% Tween 20 detergent (TBST), the membrane was incubated with horseradish peroxidase (HRP)-conjugated rabbit anti-human IgG (Sigma, USA) secondary antibody (dilution: 1:5000) for 1 h at room temperature. Next, the membranes were washed again before using ECL protein chromogenic kit (Millipore).

Nano-LC-Q-TOF-MS/MS (Sciex, USA) was used to identify the proteins detected from the serum samples of the patients with ESCC but not HC. Protein identification corresponding to protein sequences with a minimum of two unique peptides was accepted (20, 21).

### 2.3 Preparation of recombinant protein CETN2

The sequences of CETN2 were obtained from The National Center for Biotechnology Information database (www.ncbi.nlm.nih.gov). Full length of the wild-type human CETN2 (Gene ID: 1069) cDNA fragment was inserted into the pET28a vector between the NdeI-XhoI sites (Biomed, China), and then the recombinant plasmid was transformed into *Escherichia coli* BL21 (DE3) (Tiangen, China) cells. Protein expression was induced by 0.4 mM isopropyl-β-d-thiogalactoside (IPTG, Solarbio, China) at 16°C for 12 h. Following ultrasonication and high-speed centrifugation at 10000 rpm for 40 min at 4°C, the expressed recombinant protein was purified *via* affinity chromatography and then analyzed using SDS-PAGE and Coomassie brilliant blue staining. Full length of POFUT1 (Human) recombinant protein was purchased from Abnova (China).

### 2.4 Detection of serum autoantibody levels *via* ELISA

Recombinant proteins CETN2 (4 µg/mL) and POFUT1 (2 µg/mL) were diluted using a coating buffer (0.05 M carbonate/bicarbonate, pH 9.6, Solarbio), coated on 96-well microplates (Corning, USA) (100 µL/well), and incubated at 37°C for 2h followed by incubation at 4°C overnight. The plates were washed once with 0.05% Tween-20 phosphate-buffered saline (PBS, pH 7.2–7.4) and blocked and incubated for 2 h at 37^°^C with 200 µL of 10% newborn bovine serum (NBS, Gibco, USA) and 0.5% sucrose (Solarbio) diluted in PBS. Then, the plates were washed again and 50 µL of the serum sample (dilution: 1:100) in 10% NBS added to the plates and incubated for 1 h at room temperature. Next, the plates were washed thrice and incubated for 30 min at room temperature with 50 µL of HRP-conjugated rabbit anti-human IgG (Sigma) secondary antibody. The secondary antibody dilution ratio of each protein was as follows: CETN2, 1:8000; POFUT1, 1:2000. Further, after washing the plates thrice, the plates were added to 100 µL of TMB HRP substrate (Solarbio) and incubated for 15 min in the dark at room temperature to allow the reaction to occur and were stopped by adding 50 µL stop solution (Solarbio). The optical density (OD) value was recorded immediately at 450 nm using a SpectraMax M3 microplate (Thermo Fisher Scientific, USA). The OD value of each serum sample was subtracted from the OD value of its corresponding buffer-coated well in order to reduce the error caused by the non-specific combination of samples. The standard was serum mixture of selected cases with high level of autoantibody for each protein, with concentrations set to 8000 and diluted in a twofold concentration gradient to generate a standard curve for each plate. The relative levels of the two autoantibodies in each serum sample were calculated by correlating their OD value with standard curves.

### 2.5 Verification of the serum CETN2 and POFUT1 autoantibodies by western blotting

The expression levels of serum CETN2 and POFUT1 autoantibodies were verified using western blot analysis. Briefly, recombinant CETN2 protein and POFUT1 protein were electrophoresed *via* 12% SDS-PAGE and transferred to PVDF membranes. Further, after being blocked with 5% fat-free milk (BD) for 1 h at room temperature, the membrane was cut into strips and incubated with the serum samples with different CETN2 autoantibody (dilution: 1:800) and POFUT1 autoantibody (dilution: 1:1000) levels determined by ELISA assays overnight at 4°C, with CETN2 Polyclonal Antibody (dilution: 1:1000; CST, USA) and POFUT1 Polyclonal Antibody (dilution: 1:1000; Abcam, UK) as positive control, respectively. The membranes were washed thrice with TBST and the serum samples membranes were incubated with HRP-conjugated rabbit anti-human IgG (dilution: 1:5000, Sigma), and the positive control membranes were incubated with Goat anti-rabbit IgG H&L (HRP) (dilution: 1:5000, ZSGB-Bio, China) at 37°C for 1 h. The reactions were performed with an ECL protein chromogenic kit (Millipore).

### 2.6 Immunohistochemical analysis

Histological sections of tissue samples were deparaffinized through xylene and gradient alcohol. Antigens were extracted with citric acid antigen repair buffer (pH 6.0, ZSGB-Bio). The slides were incubated in 3% H_2_O_2_ solution (Kit I, ZSGB-Bio) prepared in methanol for 10 min at room temperature to block endogenous peroxidase activity, washed with PBS (pH 7.4) and blocked with blocking buffer (PBS with 10% fetal bovine serum). Then the slides were subsequently incubated with CETN2 Polyclonal Antibody (dilution: 1:100, Invitrogen, USA) or POFUT1 Polyclonal Antibody (dilution: 1:100, Abcam) overnight at 4°C. Next, the slides were rinsed and incubated with polyclonal anti-rabbit IgG secondary antibody (Kit II, ZSGB-Bio). Immunocomplexes were detected using 3,3’-diaminobenzidine (DAB, ZSGB-Bio) and staining was observed under a microscope. Finally, the slices were dehydrated and stored.

### 2.7 Statistical analysis

SPSS (version 22.0) was used for statistical analyses, and graphs were created using Graph Pad Prism 8 software. Student’s t-test was used to determine the differences in the serum autoantibody levels between the patients and controls and using nonparametric Mann–Whitney U test if the data were not normally distributed. Further, the positive rates of autoantibodies between the cancer and control groups or in the patient sera of the different stages were compared using chi-squared (χ2) tests. Receiver operating characteristic (ROC) curve was plotted to discuss the sensitivity, specificity, and the area under the curve (AUC) was used to evaluate the diagnostic performance of the candidate autoantibodies with 95% confidence intervals (95% CI). The Youden index was used to calculate the cutoff values. *P* value<0.05 was considered statistically significant.

## 3 Results

### 3.1 SERPA analysis for screening ESCC-related TAAbs

**Figure 1:**
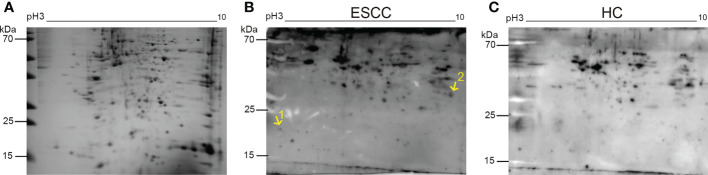
Representative two-dimensional electrophoresis map of proteins from the esophageal squamous cell carcinoma (ESCC) cell lines and the corresponding immunoblotting maps. The electrophoretic gel stained with Coomassie brilliant blue **(A)**, the immunoblotting maps of mixed serum samples from 10 ESCC patients **(B)** or 10 healthy subjects **(C)** as the primary antibody in western blot assays. The positions of the two protein spots were labeled on the 2-DE patterns. Points 1 and 2 are the sites of autoantibodies to CETN2 and POFUT1. HC, Healthy controls.

The total protein extracted from the five ESCC cell lines was lysed and divided into equal portions to conduct electrophoresis. Then, the SDS-PAGE gel was stained with Coomassie brilliant blue (**Figure 1A**). **Figures 1B, C** displayed the immunoblotting maps of the PVDF membranes for western blot assay using the mixed serum samples from 10 patients with early ESCC and 10 healthy participants as the first antibody, respectively. By comparing the immunoblots on the original 2-DE gels, the immunoblot spots that frequently existed in the cancer group, but not from control group were excised from the gels and subjected to nano-LC-Q-TOF-MS/MS. After extensive literature review and database search, the TAAbs of CETN2 and POFUT1 were identified, which were associated with tumor development (22, 23). The information of candidate TAAbs CETN2 and POFUT1 was presented in **Supplementary Table 2**, and the original MS files were provided in the online database (DOI: 10.5281/zenodo.7613741).

### 3.2 Diagnostic performance of the two candidate autoantibodies as examined *via* ELISA

**Figure 2:**
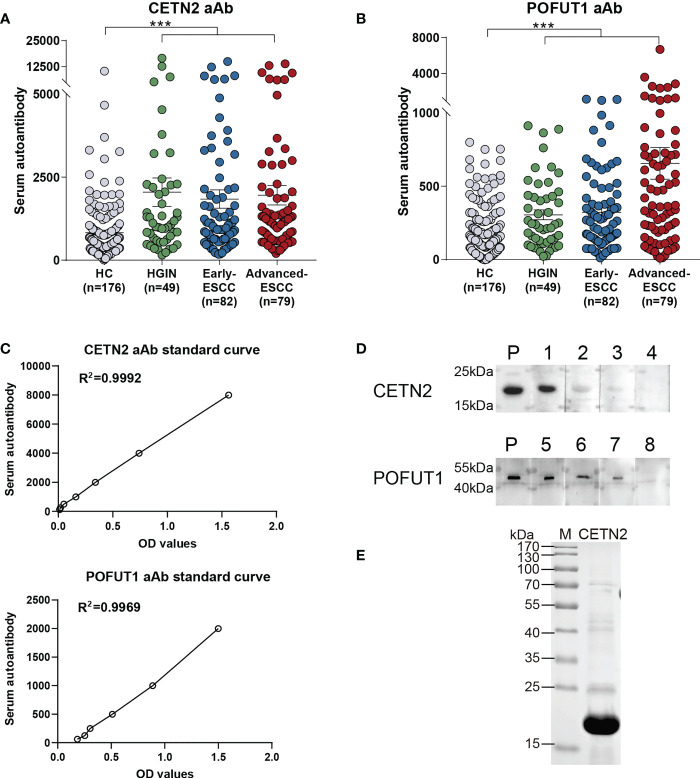
Serum autoantibody levels of CETN2 and POFUT1 as detected by enzyme-linked immunosorbent assay and verified *via* western blotting. The serum autoantibody levels of CETN2 **(A)** and POFUT1 **(B)** in the cohort. Black horizontal lines are mean with SEM; **(C)** Standard curves used for quantifying CETN2 and POFUT1 autoantibodies shown by linear regression; **(D)** Validation of the serum samples with different autoantibody levels of CETN2 and POFUT1 *via* western blot analysis. Labels 1–4 denote serum samples with concentrations of 10704.3, 6545.9, 5186.6 and 1301.2 for CETN2 aAb in ELISA analysis; Labels 5-8, denote serum samples with concentrations of 2569.3, 899.4, 490.0 and 94.5 for POFUT1 aAb in ELISA analysis, respectively. **(E)** SDS-PAGE analysis of recombinant protein CETN2. The CETN2 and POFUT1 molecular weights are approximately 20 kDa and 44 kDa, respectively. M, Marker (Thermo Fisher Scientific); P, Positive control; aAb, Autoantibody; ESCC, Esophageal squamous cell carcinoma; HGIN, High-grade intraepithelial neoplasia; HC, Healthy controls. ***p < 0.001.

Based on the results of MS, the levels of serum autoantibodies were further measured using ELISA. **Figures 2A, B** showed that the levels of CETN2 and POFUT1 autoantibodies in the cohort were increased significantly in the patients with HGIN (p<0.001) and further increased in the patients with early and advanced ESCC(p<0.001). **Figure 2C** showed the standard curves used to quantify autoantibodies to CETN2 and POFUT1.

**Figure 3:**
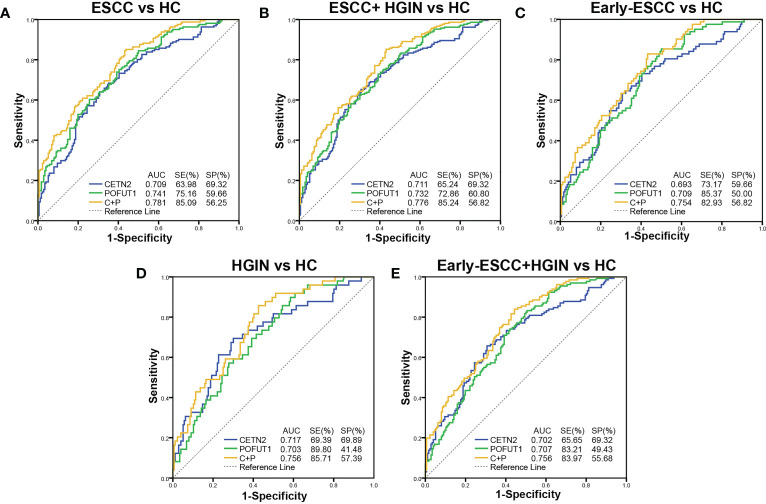
Diagnostic values of the autoantibodies of CETN2 and POFUT1 in the clinical cohort. Receiver operating characteristic (AUC) curve analysis for distinguishing between patients with esophageal squamous cell carcinoma (ESCC) from healthy controls [HC, **(A)**], patients with ESCC and high-grade intraepithelial neoplasia (HGIN) from HC **(B)**, patients with early ESCC from HC **(C)**, patients with HGIN from HC **(D)**, and patients with early ESCC and HGIN from HC **(E)**. SE, Sensitivity; SP, Specificity; C+P, Combined diagnostic value of CETN2 and POFUT1 autoantibodies.

**Figure 4:**
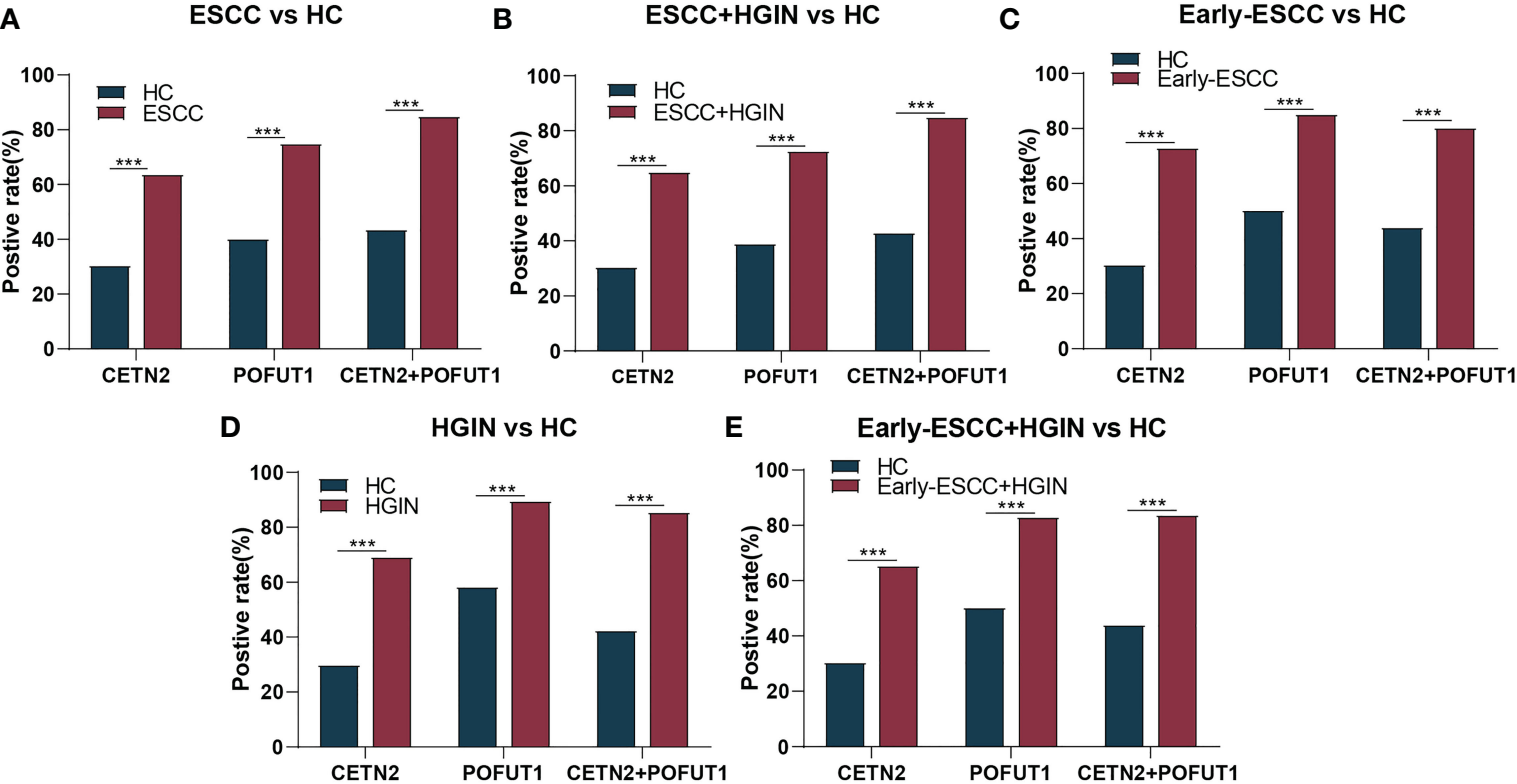
The positive rate for distinguishing between different stages of ESCC from HC. The positive rate for distinguishing between patients with esophageal squamous cell carcinoma (ESCC) from healthy controls [HC, **(A)**], patients with ESCC and high-grade intraepithelial neoplasia (HGIN) from HC **(B)**, patients with early ESCC from HC **(C)**, patients with HGIN from HC **(D)**, and patients with early ESCC and HGIN from HC **(E)**. CETN2+POFUT1, Combined diagnostic value of CETN2 and POFUT1 autoantibodies. ***p< 0.001.

ROC was used to evaluate the clinical diagnostic effect of the autoantibodies. The AUCs of each autoantibody and their combination for distinguishing the patients with ESCC from HC, patients with ESCC and HGIN from HC, patients with early ESCC from HC, patients with HGIN from HC, and those with early ESCC and HGIN from HC in the cohort were shown in **Figure 3**. The CETN2 autoantibody showed AUCs of 0.709, 0.711, 0.693, 0.717 and 0.702, sensitivities of 63.98%, 65.24%, 73.17%, 69.39% and 65.65%, and specificities of 69.32%, 69.32%, 59.66%, 69.89%, 69.32%, respectively. The POFUT1 autoantibody showed AUCs of 0.741, 0.732, 0.709, 0.703 and 0.707, sensitivities of 75.16%, 72.86%, 85.37%, 89.80%, 83.21%, and specificities of 59.66%, 60.80%, 50.00%, 41.48% and 49.43%, respectively. Remarkably, the combination of CETN2 and POFUT1 autoantibodies achieved AUCs of 0.781 (95%CI: 0.733-0.829) for distinguishing the patients with ESCC from HC (**Table 1**). The graph of positive rate was shown in **Figure 4**.

**Table 1:** The diagnostic value of CETN2, POFUT1 autoantibodies and their combination in the detection of ESCC and precancerous lesions.

	aAb	AUC	P	95%CI	Cutoff	SE (%)	SP (%)
ESCC vs HC	CETN2	0.709	<0.0001	0.654-0.764	778.98	63.98	69.32
	POFUT1	0.741	<0.0001	0.689-0.793	141.88	75.16	59.66
	CETN2+POFUT1	0.781	<0.0001	0.733-0.829	0.3273	85.09	56.25
ESCC+HGIN vs HC	CETN2	0.711	<0.0001	0.660-0.762	778.98	65.24	69.32
	POFUT1	0.732	<0.0001	0.683-0.782	145.02	72.86	60.80
	CETN2+POFUT1	0.776	<0.0001	0.730-0.821	0.3941	85.24	56.82
Early-ESCC vs HC	CETN2	0.693	<0.0001	0.623-0.762	633.12	73.17	59.66
	POFUT1	0.709	<0.0001	0.645-0.773	101.47	85.37	50.00
	CETN2+POFUT1	0.754	<0.0001	0.694-0.814	0.2281	82.93	56.82
HGIN vs HC	CETN2	0.717	<0.0001	0.634-0.800	807.62	69.39	69.89
	POFUT1	0.703	<0.0001	0.627-0.779	79.49	89.80	41.48
	CETN2+POFUT1	0.756	<0.0001	0.686-0.827	0.1541	85.71	57.39
Early-ESCC+HGIN vs HC	CETN2	0.702	<0.0001	0.643-0.761	778.98	65.65	69.32
	POFUT1	0.707	<0.0001	0.650-0.764	98.78	83.21	49.43
	CETN2+POFUT1	0.756	<0.0001	0.703-0.808	0.3121	83.97	55.68

aAb, Autoantibody; AUC, The area under the curve; 95%CI, 95% confidence interval; SE, Sensitivity; SP, Specificity; ESCC, Esophageal squamous cell carcinoma; HGIN, High-grade intraepithelial neoplasia; HC, Healthy controls; CETN2+POFUT1, Combined diagnostic value of CETN2 and POFUT1 autoantibodies.

When further combining age and gender with CETN2 and POFUT1 autoantibodies, the new prediction algorithm showed better diagnostic performance compared to the combined diagnostic value of CETN2 and POFUT1 autoantibodies, with the AUCs of 0.900 (95%CI: 0.868-0.933), 0.884 (95%CI: 0.842-0.926), 0.835 (95%CI: 0.771-0.898) in detecting ESCC, early ESCC and HGIN, respectively (**Supplementary Table 3**).

### 3.3 Comparison of autoantibody positive rates of CETN2 and POFUT1 in patients and those of conventional clinical serological markers

**Figure 5:**
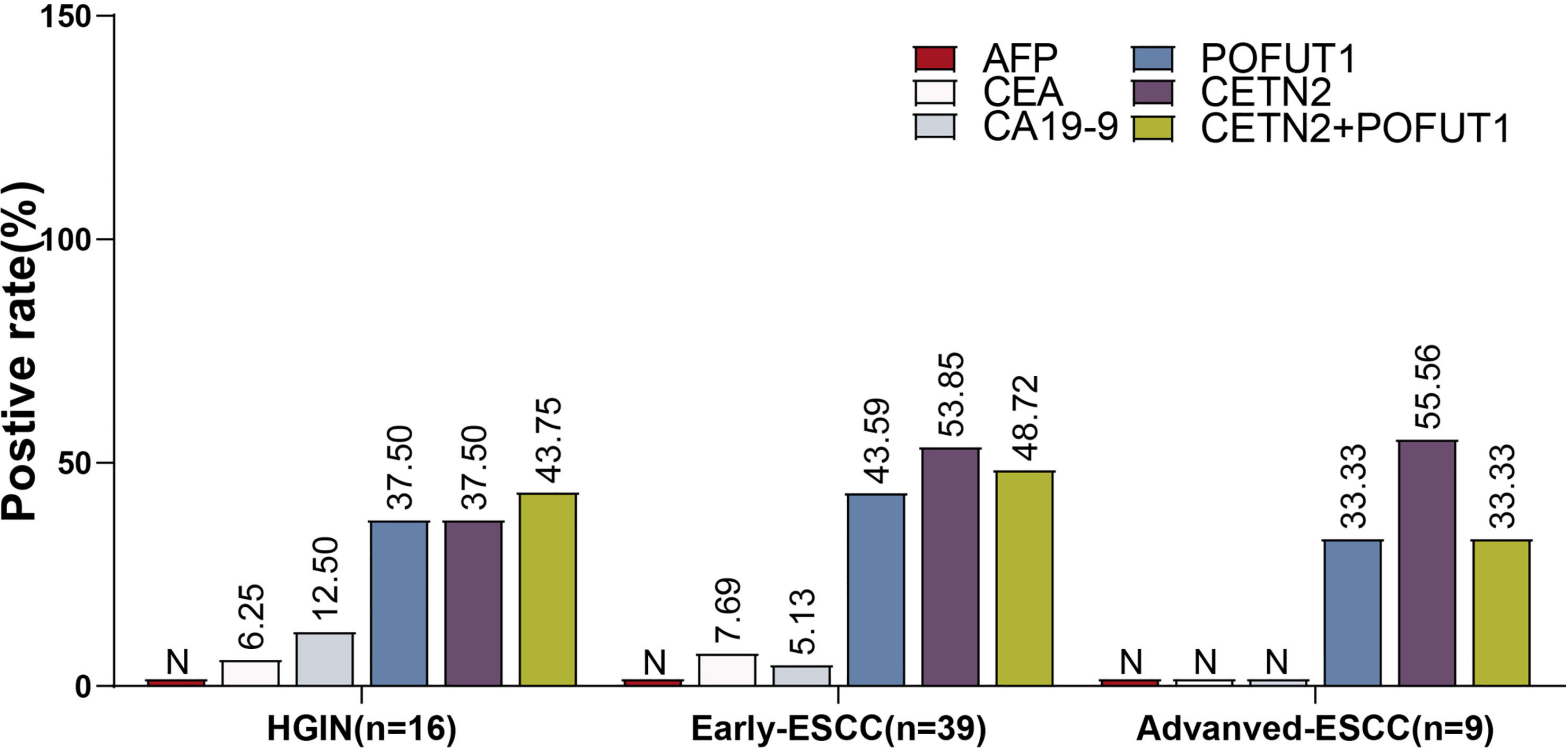
Comparison of the positive rates of CETN2 and POFUT1 autoantibodies with those of conventional clinical serological markers for esophageal squamous cell carcinoma (ESCC) or high-grade precancerous lesions (HGIN). All patients were negative for alpha-fetoprotein (AFP), and the patients with advanced ESCC were also negative for carcinoembryonic antigen (CEA) and carbohydrate antigen 19-9 (CA 19-9). N, Negative; CETN2+POFUT1, Combined diagnostic value of CETN2 and POFUT1 autoantibodies.

We used the clinically defined range (AFP:0-15 ng/mL; CEA: 0-5 ng/mL; CA19-9: 0-35 U/mL) to determine the positive rates of AFP, CEA and CA19-9. Further, in those patients whose information regarding tumor markers could be obtained, the AFP levels were all negative. Based on the cutoff value determined by 80.1% specificity, the positive rates of CEA, CA19-9 and the autoantibodies of POFUT1 and CETN2 were compared (**Figure 5**). As a result, the positive rates of POFUT1 autoantibody (37.50%, 43.59%, 33.33%) and CETN2 autoantibody (37.50%, 53.85%, 48.72%) in HGIN, early ESCC, and advanced ESCC were higher than those of the other clinical markers. Meanwhile, the highest positive rate of CEA and CA19-9 was only 12.50%, while the positive rate of combined CETN2 and POFUT1 autoantibodies was more than 33.33% in different stages, implying the potential value of CETN2 and POFUT1 autoantibodies for early screening and detection of early ESCC.

### 3.4 Confirmation of the different levels of serum CETN2 and POFUT1 autoantibodies *via* western blot analysis

Western blotting with recombinant CETN2 and POFUT1 protein was conducted to measure the levels of CETN2 and POFUT1 autoantibodies in the different serum samples (**Figure 2D**). SDS-PAGE analysis of recombinant CETN2 protein was shown in **Figure 2E**. The serum samples with different concentrations of CETN2 and POFUT1 autoantibodies detected by ELISA were selected as primary antibodies and verified by western blot analysis, respectively. The levels were found to be consistent with those detected *via* ELISA.

### 3.5 CETN2 and POFUT1 are overexpressed in human esophageal squamous cell carcinoma

**Figure 6:**
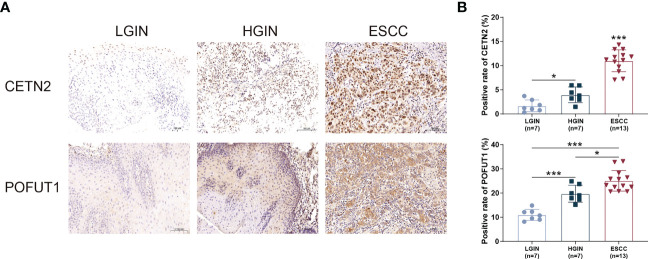
Immunohistochemical staining of tissues at various stages of ESCC and precancerous lesions. **(A)** Immunohistochemical staining of tissue sections at various stages. Magnification: ×20; **(B)** Comparison of the CETN2 and POFUT1 expression in patients with ESCC and precancerous lesions. ESCC, Esophageal squamous cell carcinoma; HGIN, High-grade intraepithelial neoplasia; LGIN, Low-grade intraepithelial neoplasia. *p< 0.05; ***p< 0.001.

CETN2 and POFUT1 immunolabeling was performed in tissues representing ESCC and precancerous lesions, which confirmed that the expression of CETN2 and POFUT1 increased with the severity of ESCC. The levels of CETN2 and POFUT1 in tissues of ESCC and HGIN patients were higher than that in LGIN (**Figure 6**).

## 4 Discussion

The screening and diagnosis of early ESCC and precancerous lesions are critical for preventing the further worsening of the condition and providing effective treatment. Presently, conventional endoscopy is used as a method for detecting early ESCC, which is an invasive technique, and there are no specific noninvasive methods for the same purpose. Several antigen-based serum biomarkers for early ESCC have been explored to be used as noninvasive diagnostic methods; however, these biomarkers possess reduced sensitivity and high specificity, thereby rendering them unsuitable for clinical diagnosis (9). Recently, serum TAAb biomarkers have been recognized as promising for the early screening of cancers. In the present study, *via* an initial screening using SERPA combined with nano-LC-Q-TOF-MS/MS and further validation through ELISA, we identified novel autoantibody biomarkers of CETN2 and POFUT1. These biomarkers may have diagnostic value for early ESCC and precancerous lesions and provide new insights for the detection of early ESCC.

Evaluation of diagnostic value of autoantibody-based biomarkers has shown promising value in clinical application (24). Chu et al. found that the levels of seven autoantibodies were significantly higher in early-stage oral cavity squamous cell carcinoma (OSCC) patients than in healthy individuals (25). Hsueh et al. screened diagnostic autoantibody-based markers for OSCC, and found that the sensitivity and specificity for early OSCC detection by using marker panels composed of MMP3, PRDX2, SPARC, and HSPA5 was 63.8% and 90% (26). Yang et al. screened potential autoantibody-based markers for hepatocellular carcinoma (HCC) and found that the AUC of an immunodiagnostic model including 6 TAAbs (RAD23A, CAST, RUNX1T1, PAIP1, SARS, PRKCZ) were 0.835 and 0.788 in the training and validation sets, respectively (27). Wang et al. found that the model comprising 4 TAAbs and CEA reached an AUC of 0.813 for diagnosing patients of lung cancer from normal individuals (28). At present, more than 100 autoantibodies have been used in early detection studies of female breast cancer, and the panel sensitivity values reported across studies were higher with an estimated range of 60-87% (29). However, Zhang et al. performed a meta-analysis on the diagnostic value of anti-p53 antibody as a single biomarker in esophageal cancer and reported a dissatisfactory sensitivity range of 15%–60% with a specificity range of 91%–100%. This shows that, there were large-scale fluctuations between small samples and different studies (30). Moreover, some studies have found that there is no significant difference in p53 autoantibodies between cancer and non-cancer sera (31, 32). Xu et al. employed two independent cohorts to study a panel of six autoantibodies against p53, NY-ESO-1, MMP-7, HSP70, PRDX-6, and BMI-1 and found that a single cancer-associated autoantibody biomarker has limited diagnostic value (33). Additionally, other than the studies of Zhang et al. and Xu et al., there remains a lack of diagnostic evaluation of patients with early ESCC and precancerous lesions, yet diagnosis in the early stages of cancer is often most important to reduce mortality.

In the current study, based on the clinical resource of early ESCC and HGIN, we were able to initially screen specific autoantibody biomarkers for the detection of early ESCC *via* SERPA combined with nano-LC-Q-TOF-MS/MS, and two potential autoantigens including CETN2 and POFUT1 were frequently recognized by the serum of early ESCC patients. Meanwhile, there may exist other candidate TAAbs which were not included in the present study. Furthermore, to evaluate the diagnostic value of the initially screened autoantigens for early ESCC and precancerous lesions, we employed ELISA to detect the serum levels of two autoantibodies in patients with ESCC, patients with HGIN and HC. The results revealed that the levels of CETN2 and POFUT1 autoantibodies were significantly elevated in patients with HGIN and ESCC.

To the best of our knowledge, there have been no reports on the relationship between CETN2 and POFUT1 autoantibodies and esophageal cancer. Understanding the mechanisms of these autoantibodies is essential for their clinical application. The overexpression of TAAs is one of the mechanisms of autoantibody production (34). CETN2 is a ubiquitous protein component present in the centrosome and polar body of the mitotic spindle, and abnormalities in centrosome replication are prevalent in various cancers, which are already occasionally present in precancerous lesions (35). POFUT1 is an essential enzyme that catalyzes the synthesis of protein O-fucosylation (36), which has been validated and can be applied as a diagnostic marker of colorectal cancer (37). Moreover, there have been several reports regarding abnormal fucosylation in cancers, such as lung, colorectal, breast, ovarian, liver, and pancreatic cancers (38, 39). In our study, the expression of CETN2 and POFUT1 was found to be positively correlated with ESCC progression, which may have directly led to an immune response that stimulated the production of autoantibodies. Although the underlying mechanism needs to be further explored, these findings provide support for the elevated levels of CETN2 and POFUT1 autoantibodies in precancerous lesions and their application in the clinical diagnosis of early ESCC.

However, the current study has certain limitations. First, it is difficult to collect patients’ serum with early lesions owing to the difficulty of diagnosis, leading to an insufficient number of cases in the current cohort. We anticipated validating the significant findings of the present study in a larger sample cohort in the future, and conduct further evaluation by expanding the sample size of the healthy participants and measuring the normal reference range as cutoff values. Second, as the HC were not conducted endoscopic examination, we cannot exclude the possibility that there were no early-stage lesions in HC, and the diagnostic value of the autoantibody markers may be improved if HC without early-stage lesions after endoscopy examination is more strictly included as a control. In addition, the CA19-9,CEA and AFP values were obtained from clinical records for some patients after their diagnosis of ESCC or HGIN and missing data existed for some cases, which may result in bias in the comparison of the diagnostic performance of the conventional tumor serum markers and autoantibodies, which would be further verified by future studies. Based on the above results, we will conduct further evaluation of ESCC-related TAAbs in the future.

## 5 Conclusions

Our study revealed that CETN2 and POFUT1 autoantibodies have potential clinical diagnostic value for early ESCC and precancerous lesions, and the detection of serum autoantibodies of CETN2 and POFUT1 can improve the diagnostic rate of early ESCC. Although performed in small cohorts, these results offer potential insights into the detection of early ESCC and precancerous lesions and offer new serum autoantibody biomarkers for early ESCC diagnosis. Moreover, it could be used as a complement to other non-invasive markers for ESCC screening test

## Data availability statement

The datasets presented in this study can be found in online repositories. The names of the repository/repositories and accession number(s) can be found below: 10.5281/zenodo.7613741.

## Ethics statement

This study was approved by the Ethics Committee of Beijing Friendship Hospital, Capital Medical University (No.2018-P2-058-01) and Cancer Hospital, Chinese Academy of Medical Sciences and Peking Union Medical College (No.16-171/1250). The patients/participants provided their written informed consent to participate in this study. All procedures performed in studies involving human participants were in accordance with the ethical standards of the institutional and national research committee and with the Code of Ethics of the World Medical Association (Declaration of Helsinki). Informed consent was obtained from all subjects involved inthe study.

## Author contributions

JH and SZ: Conceptualization Ideas, Methodology, Supervision; ZC, QZ, PH: Investigation, Formal analysis, Writing – original draft; JX and CZ: Data Curation, Writing – original draft; SQ, QO, XL, BZ: Formal analysis. DZ and YL: Writing - Review & Editing. YX, JR, BC: Resources, Data Curation. All authors contributed to the article and approved the submitted version. 
