# Supplementary material for: Identification of novel serum autoantibody biomarkers for early esophageal squamous cell carcinoma and high-grade intraepithelial neoplasia detection
Source: Front Oncol. 2023 May 12;13:1161489. doi: 10.3389/fonc.2023.1161489 (PMC10213680; doi:10.3389/fonc.2023.1161489)
Supplement: Supplementary file 1 [file Table_1.pdf]

Supplementary Material

Identification of novel serum autoantibody biomarkers for early esophageal squamous cell carcinoma and high-grade intraepithelial neoplasia detection

Zhibin Chen†, Jie Xing†, Cuiling Zheng, Qianyu Zhu, Pingping He, Donghu Zhou, Xiaojin Li, Yanmeng Li, Saiping Qi, Qin Ouyang, Bei Zhang, Yibin Xie, Jiansong Ren, Bangwei Cao, Shengtao Zhu*, Jian Huang*

*** Correspondence:**
Jian Huang*: huangj@ccmu.edu.cn
Shengtao Zhu*: zhushengtao@ccmu.edu.cn

Supplementary Tables

Supplementary Table 1. Demographic parameters of patients and healthy control of the research subjects.

		Serums for SERPA		Clinical cohort for ELISA				Tissues for IHC		
Variables		Healthy control (n=10)	Early-ESCC (n=10)	Healthy control (n=176)	HGIN (n=49)	Early-ESCC (n=82)	Advanced-ESCC (n=79)	LGIN (n=7)	HGIN (n=7)	ESCC (n=13)
Age										
	Median (Min-Max)	64.00 (55-77)	63.50 (49-74)	63.50 (22-80)	65.00 (48-92)	62.00 (42-86)	65.00 (34-92)	57.00 (46-80)	62.00 (53-73)	63.00 (42-80)
	Interquartile range (IQR)	9.00	12.50	18.00	16.50	9.25	10.00	18.00	13.00	16.50
	Mean	63.70	62.60	62.20	63.80	62.74	64.13	58.86	63.00	63.77
	Std. Deviation	6.99	7.68	13.49	9.74	8.06	8.64	11.80	7.48	11.34
Gender	(Male/Female)	4/6	8/2	54/122	32/17	64/18	69/10	3/4	5/2	10/3

Supplementary Table 2. Identification of CETN2 and POFUT1 proteins by Nano-LC-Q-TOF-MS/MS.

Spot number	Protein	Accession	Coverage (95%)	Peptides matched (95%)
1	Centrin-2 OS=Homo sapiens GN=CETN2 PE=1 SV=1	sp|P41208|CETN2_HUMAN	34.30	5
2	GDP-fucose protein O-fucosyltransferase1 OS=Homo sapiens GN=POFUT1 PE=1 SV=1	sp|Q9H488|OFUT1_HUMAN	7.90	3

Supplementary Table 3. The combined diagnostic value of CETN2 and POFUT1 autoantibodies, age and gender in the detection of ESCC and precancerous lesions.

	AUC	P	95%CI	Cutoff	SE (%)	SP (%)
ESCC vs HC	0.900	<0.0001	0.868-0.933	0.4759	85.71	81.82
ESCC+HGIN vs HC	0.884	<0.0001	0.851-0.917	0.4541	85.24	77.84
Early-ESCC vs HC	0.884	<0.0001	0.842-0.926	0.3495	85.37	78.41
HGIN vs HC	0.835	<0.0001	0.771-0.898	0.2073	75.51	77.84
Early-ESCC+HGIN vs HC	0.865	<0.0001	0.825-0.905	0.4345	78.63	80.11
